# *Cmcrf1*, a Putative Zn2Cys6 Fungal Transcription Factor, Is Involved in Conidiation, Carotenoid Production, and Fruiting Body Development in *Cordyceps militaris*

**DOI:** 10.3390/biology11101535

**Published:** 2022-10-19

**Authors:** Ronglin He, Lin Zhang, Jinling Lan, Shengjie Mei, Yu Li

**Affiliations:** 1 Tianjin Institute of Industrial Biotechnology, Chinese Academy of Sciences, Tianjin 300308, China; 2 National Center of Technology Innovation for Synthetic Biology, Tianjin 300308, China; 3 College of Plant Protection, Jilin Agricultural University, Changchun 130118, China

**Keywords:** T-DNA insertional mutant, fungal development, carotenoid biosynthesis, edible and medicinal fungi

## Abstract

**Simple Summary:**

*Cordyceps militaris* produce a wide variety of bioactive components, such as cordycepic acid, cordycepin, polysaccharides, pentostatin, ergosterol, and carotenoids. In particular, natural carotenoids from *C. militaris* are attracting increasing attention in human healthy and food coloring. Investigating the genetic regulatory mechanism of carotenoid biosynthesis will help to increase the carotenoid content of *C. militaris* through genetically engineering. This study focuses on the role of a putative Zn_2_Cys_6_ fungal transcription factor *Cmcrf1* on carotenoid biosynthesis and fruiting body formation. Deletion of *Cmcrf1* exhibited drastically reduced carotenoid biosynthesis and failed to generate fruiting bodies. In addition, the *ΔCmcrf1* mutant exhibited significantly increased conidiation and increased hypersensitivity to cell-wall-perturbing agents. This study is helpful to deepen our knowledge of the regulatory mechanism of carotenoid biosynthesis in *C. militaris*.

**Abstract:**

*Cordyceps militaris* is a high-value medicinal and edible fungus that produces many bioactive compounds, including carotenoid, and thus, improving the carotenoid productivity of *C. militaris* will increase its commercial value. However, little is known about the genetic regulatory mechanism of carotenoid biosynthesis in *C. militaris*. To further understanding the regulatory mechanism of carotenoid biosynthesis, we performed a large-scale screen of T-DNA insertional mutant library and identified a defective mutant, denoted T111, whose colonies did not change color from white to yellow upon exposure to light. Mutation analysis confirmed that a single T-DNA insertion occurred in the gene encoding a 695-amino-acid putative fungal-specific transcription factor with a predicted Zn_2_Cys_6_ binuclear cluster DNA-binding domain found uniquely in fungi. Targeted deletion of this gene, denoted *C. militaris* carotenogenesis regulatory factor 1 (*Cmcrf1*), generated the *ΔCmcrf1* mutant that exhibited drastically reduced carotenoid biosynthesis and failed to generate fruiting bodies. In addition, the *ΔCmcrf1* mutant showed significantly increased conidiation and increased hypersensitivity to cell-wall-perturbing agents compared with the wild-type strain. However, the *Cmcrf1* gene did not have an impact on the mycelia growth of *C. militaris*. These results show that *Cmcrf1* is involved in carotenoid biosynthesis and is required for conidiation and fruiting body formation in *C. militaris*.

## 1. Introduction

*Cordyceps militaris* is an entomopathogenic fungus that parasitizes the larvae or pupae of lepidopteran insects. It is also an edible fungus with medicinal properties and has been widely used in traditional Chinese medicine as a substitute for *C. sinensis*, as both species produce a variety of similar bioactive ingredients. *C. militaris* produce a wide variety of bioactive components, such as cordycepic acid, cordycepin, polysaccharides, pentostatin, ergosterol, and carotenoids [1], compounds that possess anti-fatigue, anti-tumor, anti-oxidant, and anti-inflammatory activities [2]. Particular attention has been paid to the carotenoids produced by *C. militaris* due to their strong antioxidant activities [3].

Natural carotenoids are widely used in health food industries. More than 700 natural carotenoids have been identified so far [4]. *C. militaris* has in recent years been investigated for carotenoid production [5].The productivity of carotenoid in *C. militaris* is relatively low and cannot meet the market demands. With the rapid development of synthetic biology, genetic engineering is an effective method to increase the carotenoid productivity of *C. militaris* [6,7,8]. However, the insufficient understanding of the carotenoid biosynthesis pathways in *C. militaris* prevents efficient genetic engineering of this fungus [9,10,11].

Most studies have focused on elucidating the key genes involved in the various metabolic pathways that affect carotenoid biosynthesis in *C. militaris*. For example, recent studies have revealed that *Cmhyd1*, which encodes a class II hydrophobin, may influence the yellow pigment accumulation in *C. militaris*. In particular, a *Cmhyd1*-null mutant formed white colonies on potato dextrose agar (PDA) plates even after light irradiation [12]. Moreover, *Cmfhp* encodes flavohemoprotein, a nitric oxide (NO) dioxygenase, that was found to significantly affect carotenoid biosynthesis, as the carotenoid content of the *Cmfhp*-null mutant decreased compared with the WT strain in *C. militaris* [13]. *Cmtns* encoding a terpenoid synthase gene was reported to have a similar effect as *Cmfhp* on the carotenoid production of *C. militaris* [14]. Defective primordium and fruiting bodies were also found in the null mutants of these three genes. Furthermore, high concentrations of carotenoids accumulate in the mycelia and fruiting bodies of *C. militaris* upon exposure to light during cultivation. Seven photoreceptors related to photobiological processes have been identified through genome-wide analysis of *C. militaris*, and the functions of three of these seven photoreceptors (CmWC-1, CmVVD, and CmCry-DASH) have been comprehensively investigated in *C. militaris*. Deletion of *Cmwc-1* led to a drastically decrease in carotenoid production and defects in fruiting body development [15]. Deletion of *Cmvvd*, which encodes a blue-light receptor, led to abnormal development of fruiting bodies and a significant increase in carotenoid content [16]. In contrast to the phenotypes of *ΔCmwc-1* mutant, the *Cmcry-DASH*-null mutant exhibited more carotenoid accumulation than the WT strain. However, despite the *ΔCmcry-DASH* strain exhibiting normal primordium formation, its fruiting bodies did not elongate normally [17]. In addition, although several studies have performed genome-wide transcriptional analyses and genome-level metabolic network-driven analyses to probe carotenoid biosynthetic pathways in *C. militaris*, only a few differentially expressed genes (DEGs) that might be involved in carotenoid biosynthesis have been identified [18,19]. The functions of these DEGs in *C. militaris* have yet to be elucidated, and thus, delineating the functions of genes involved in carotenoid biosynthesis in this fungus remains an important research topic.

T-DNA insertion mutation is an effective method to identify novel genes participated in the different metabolic pathways. Many novel pathogenicity-related genes have been discovered through large-scale screening of T-DNA mutant libraries of phytopathogenic fungi, such as *Verticillium dahliae*, *Ustilaginoidea virens*, and *Sporisorium scitamineum* [20,21,22]. In the industrial cultivation of filamentous fungi, the ATMT method has been proven an efficient tool for screening random T-DNA insertion mutants to identify those with improved production of certain metabolic products [23,24]. In the current study, we performed a large-scale screen of T-DNA tagged mutants of *C. militaris* and found that the mutation of a gene encoding a fungal-specific transcription factor resulted in a white colony phenotype upon exposure to light. This gene, which we designated *C. militaris* carotenogenesis regulatory factor 1 (*Cmcrf1*), encodes a 695-amino-acid protein with a predicted Zn_2_Cys_6_ binuclear cluster DNA-binding domain, demonstrating that *Cmcrf1* is a Zn_2_Cys_6_ transcription factor. We also functionally characterized *Cmcrf1* in *C. militaris* using a targeted gene-deletion strategy. Effects of *Cmcrf1* on fungal growth, conidiation, cell wall integrity, fruiting body formation, and carotenoid production were extensively investigated. This study might shed light on the regulatory mechanisms involved in carotenoid biosynthesis and fruiting body development in *C. militaris.*

## 2. Materials and Methods

### 2.1. Strains, Media, and Cultivation Conditions

*C. militaris* strain CM20-Xi (Edible Fungi Research Institute, Ankang Academy of Agricultural Sciences, Shaanxi Province) purified by single-spore isolation was used in all experiments. All of the *C. militaris* strains used or generated in this study were cultivated on modified PDA medium (20.0% potato, 1.0% dextrose, 0.1% KH_2_PO_4_, 0.05% MgSO_4_, peptone 0.2%, and 1.5% agar, *w*/*v*) at 25 °C for 20 days. For long-term conservation, strains were maintained as stocks on wheat seed medium at 4 °C. *Agrobacterium tumefaciens* was cultivated in Luria–Bertani (LB) broth or LB agar. *Escherichia coli* DH-5α was used for plasmid constructions.

### 2.2. Disruption of *Cmcrf1* and Transformation of C. militaris

*Cmcrf1* was knocked out using a homologous recombination gene-deletion strategy. Briefly, the targeted gene-deletion cassettes were constructed using a double-joint polymerase chain reaction (PCR) method [25]. The 5′- and 3′-flanking sequences of *Cmcrf1* were amplified from the genomic DNA of the WT strain with primers CRF15F/CRF15R and CRF13F/CRF13R, respectively, the sequences of which are provided in Table S1. The hygromycin B phosphotransferase gene (*hph*) was generated by PCR from the pBSHPH1 vector using the primer pair hphF/hphR [26]. All of the generated PCR products were purified using a gel extraction kit (Omega Bio-tek, USA) for the subsequent procedure. The 5′-flanking fragment, the *hph* gene, and the 3′-flanking fragment were fused together in this order, and the resulting fused deletion cassettes were then cloned into the binary vector pCAMBIA1300 to produce the targeted gene-deletion vector pCAMBIA1300-yprKO (Figure S1A). The constructed vectors were then transformed into *C. militaris* through the *Agrobacterium tumefaciens*-mediated transformation (ATMT) method as previously described, with some modifications [27]. For Southern blotting analysis, Genomic DNA extraction and restriction enzyme digestion were performed as described [28]. The 3′ flanking sequence of *Cmcrf1* was amplified from genomic DNA and labeled as a probe. Hybridization processes were conducted following the manufacturer’s instructions.

### 2.3. Complementation of the *Cmcrf1*-Null Mutant

The *Cmcrf1*-null mutant was complemented with *Cmcrf1* from the WT strain. The complementation gene sequences including the native promoter region, the full-length DNA sequence of *Cmcrf1*, and the 0.5 kb downstream sequence were amplified from the genomic DNA of WT and cloned into pCAMBIA3301 to generate the complementary vector pCAMBIA3301-yprC (Figure S1B). This vector was then transformed into the null mutant using the ATMT method. All the obtained transformants were screened on PDA with 300 μg/mL glufosinate ammonium. The complemented transformants were verified through PCR and quantitative reverse transcription (qRT)-PCR.

### 2.4. Fungal Development Assays

Mycelia disks were inoculated onto PDA plates and incubated at 25 °C for 3 weeks. The diameters of colonies were measured every 2 days. For conidial production, cultivated mycelia from 14-day-old plates were removed with a sterilized spreader and added into 50 mL centrifuge tubes containing 5 mL double-distilled (dd) H_2_O. The resulting conidial suspensions were filtered through four layers of lens paper, and the numbers of conidia in the filtered suspensions were counted with a hemocytometer under a microscope and then diluted to appropriate concentrations. For conidial germination, 10 μL droplets of the conidial suspensions (resuspended and diluted to 1 × 10^5^/mL with sterilized dd H_2_O) were placed on glass slides. The droplet-bearing slides were then incubated in a moisture chamber at 25 °C, and the conidial germination rates were examined under a microscope at 12, 14, and 16 h post inoculation (hpi). Three independent experiments were performed with three replicates each time.

### 2.5. Cell Wall Integrity Assays

Mycelial disks were inoculated onto minimal medium (MM) plates containing various chemicals, such as Calcofluor White (CFW) and Congo Red (CR), for sensitivity tests on these chemicals. The resulting inoculated plates were incubated at 25 °C for 20 days, and the diameters of colonies were examined to enable comparison between all the tested strains. The test of each strain’s sensitivity to each chemical was performed in triplicate.

### 2.6. Determination of Carotenoids

Mycelia from cultures of different strains were filtered, lyophilized, and used for carotenoid determination using the acid-heating method, as described previously [29]. Determination of the carotenoid content was performed by measuring the absorbance at 445 nm.

### 2.7. Quantitative Real-Time PCR (qRT-PCR)

Total RNA of mycelia was extracted using TRIzol reagent (Invitrogen Life Technologies, USA) and treated with DNase. All qRT-PCR assays were performed as described [30]. Three biological replicates were carried out with three technical replicates.

## 3. Results

### 3.1. Identification and Expression Pattern of T-DNA-Tagged *Cmcrf1* in C. militaris

**Figure 1:**
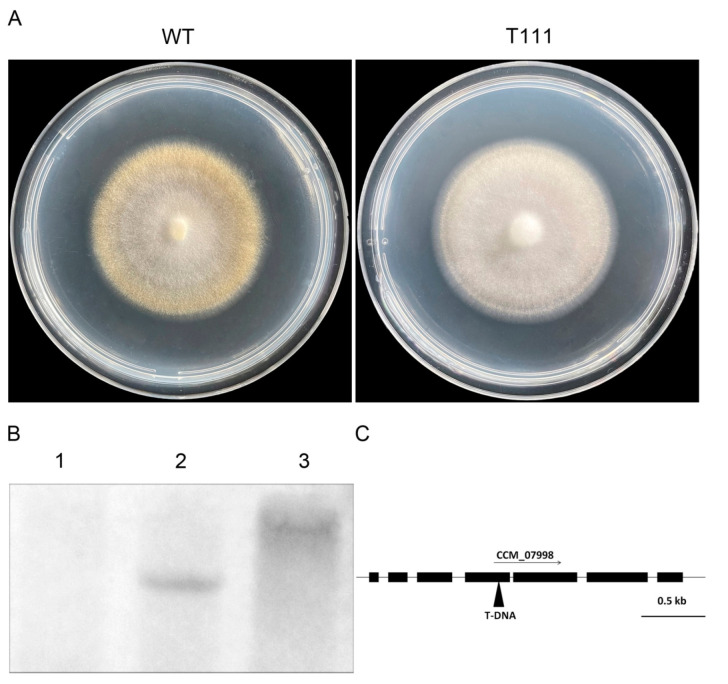
Identification of carotenogenesis-defective mutant T111 and the location of T-DNA insertion in this mutant. (**A**) Morphology of the WT strain and T-DNA insertion mutant T111 on PDA plates. (**B**) Southern blotting of the WT strain and the T111 mutant. Lane 1, genomic DNA of the WT strain digested with *Sal*I; lanes 2–3, genomic DNA of the T111 mutant digested with *Sal*I and *Hin*dIII. Genomic DNA was hybridized with the digoxigenin (DIG)-labeled 1.4 kb hygromycin resistance gene probe. (**C**) Positions of T-DNA insertions in the T111 mutant. Filled arrows indicated the positions T-DNA insertion in the predicted gene *CCM_07998*. Filled boxes represent the coding regions, and the thin line connecting these filled boxes indicates the introns.

We constructed a T-DNA insertion-mutant library of the wild-type *C. militaris* using a binary vector on PDA plates, which afforded mutants with abnormal morphology. A mutant that did not form yellow-pigmented colonies when exposed to light was picked out for further functional analysis and was designated as T111 (Figure 1A). To investigate whether single T-DNA insertion caused the mutation in the T111 mutant, the genomic DNA of T111 was digested with *Sal*I and *Hin*dIII for Southern blotting. The result showed that a single-copy T-DNA was integrated into the genome of the T111 mutant (Figure 1B). High-efficiency thermal asymmetrical interlaced PCR (hiTAIL-PCR) was performed to amplify the genomic DNA sequences flanking the integrated T-DNA [31]. Comparison between the sequences obtained by hiTAIL-PCR and the related sequences in the *C. militaris* genome database showed that the T-DNA insertion in the T111 mutant was located in the fourth exon of the predicted gene, *CCM_07998* (Figure 1C), which was annotated to encode a fungal-specific transcription factor in the database. This gene encodes a putative fungal transcription factor containing a Zn_2_Cys_6_ binuclear cluster DNA-binding domain found exclusively in fungi and a middle homology domain (Figure S2A). Thus, this gene was designated *Cmcrf1* due to the T111 mutant exhibiting defective carotenoid production. Alignment of *Cmcrf1* with its homologs from other fungal species revealed that *Cmcrf1* had 63.2%, 41.1%, 38%, and 36.9% similarities with homologs from *Akanthomyces lecanii*, *Neurospora crassa*, *Aspergillus terreus*, and *Metarhizium robertsii*, respectively (Figure S2B). Figure S1C showed the results of the phylogenetic analysis of the putative homologs of *Cmcrf1* from several filamentous fungal species. The results indicate that *Cmcrf1* is most closely related to the homologs expressed in *A. lecanii* and *N. crassa* and that it is highly conserved in filamentous fungi (Figure S2).

**Figure 2:**
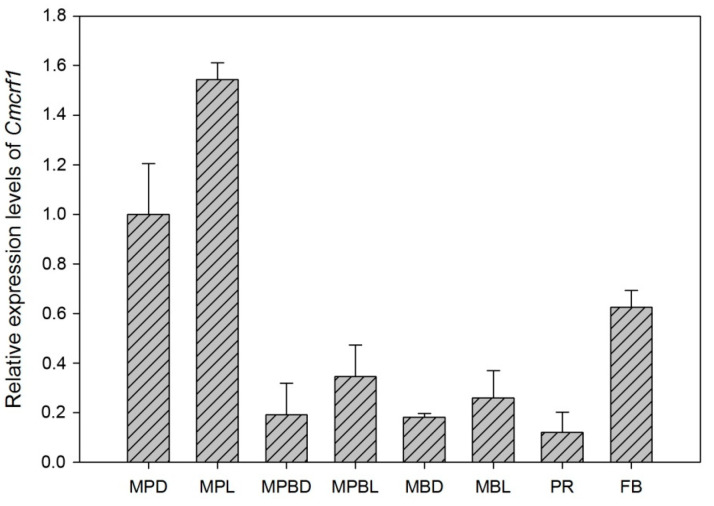
Transcription profiles of *Cmcrf1* in different stages of fungal cultivation. MPD, mycelia grown on PDA in the dark; MPL, mycelia grown on PDA in the dark and then exposed to light for 12 h; MPBD, mycelia grown in potato dextrose broth (PDB) in the dark; MPBL, mycelia grown in PDB in the dark and then exposed to light for 12 h; MBD, mycelia grown in a bottle in the dark; MBL, mycelia grown in a bottle in the dark and then exposed to light for 12 h; PR, primordium; FB, fruiting body. *Tef1* expression was used for normalization. Three biological replicates were carried out with three technical replicates. The values reported are the averages of three independent experiments ± standard deviations.

To investigate the expression pattern of *Cmcrf1*, the transcription profiles of *Cmcrf1* in different developmental stages were analyzed. The results of qPCR showed that *Cmcrf1* was constitutively expressed during the entire fungal-cultivation process (Figure 2). *Cmcrf1* expression under light exposure was higher than that under dark conditions, demonstrating that light promotes its expression. It was noteworthy that the levels of *Cmcrf1* expression were higher in the plate cultivation and fruiting body stages than in the other stages, which implies that *Cmcrf1* might play a role in vegetative growth on plates and in fruiting body development.

### 3.2. Disruption of *Cmcrf1*

**Figure 3:**
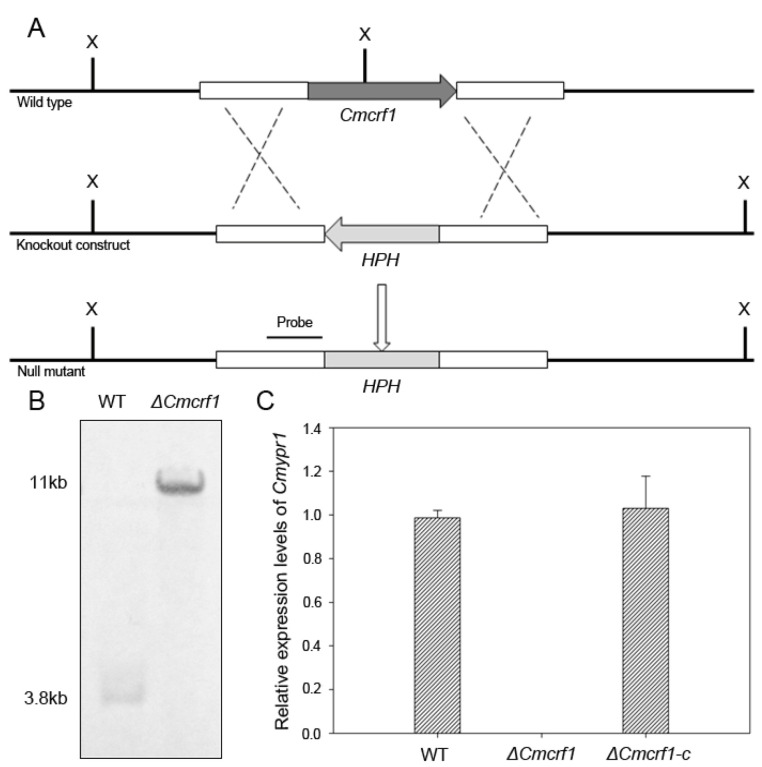
Disruption of *Cmcrf1*. (**A**) Targeted deletion of *Cmcrf1*. X, *Xba*I. (**B**) Southern blotting analysis of the WT strain and the *ΔCmcrf1* mutant. All Genomic DNA samples were digested with *Xba*I and probed with a 5′ flanking sequence of *Cmcrf1* labeled by digoxigenin. (**C**) qRT-PCR analysis of *Cmcrf1* in the WT strain, the *ΔCmcrf1* mutant, and the complemented mutant *ΔCmcrf1c*. *Cmcrf1* expression was not detected in the *ΔCmcrf1* mutant.

To characterize the function of *Cmcrf1* in *C. militaris*, a targeted gene-deletion strategy was applied (Figure 3A). Thus, the targeted gene-deletion vector pCAMBIA1300-yprKO was transformed into the WT strain using the ATMT method. All of the hygromycin-B-resistant transformants were screened by PCR using primers CRF1checkF/R. The suspected mutant was confirmed to be the *Cmcrf1* null (*ΔCmcrf1*) mutant by Southern blot analysis. The hybridized 3.8 kb band and an 11 kb band were detected in the *ΔCmcrf1* mutant and the WT strain, respectively (Figure 3B). The *Cmcrf1* mRNA expression was analyzed by qRT-PCR in and the *ΔCmcrf1* mutant. The results revealed that there was no *Cmcrf1* expressed by the *ΔCmcrf1* mutant (Figure 3C), and this confirmed *ΔCmcrf1* strain was used for further investigations.

For the generation of complemented transformants, the constructed complementary vector pCAMBIA3301-yprC was transformed into the *ΔCmcrf1* mutant. The complemented mutants grown on PDA containing glufosinate ammonium were identified by PCR and qRT-PCR. The qRT-PCR results showed that *Cmcrf1* was expressed in the WT strain and in the complemented mutant *ΔCmcrf1c*, indicating that *Cmcrf1* was successfully complemented in the *ΔCmcrf1* mutant (Figure 3C).

### 3.3. Deletion of *Cmcrf1* Affects the Growth Characteristics of C. militaris

**Figure 4:**
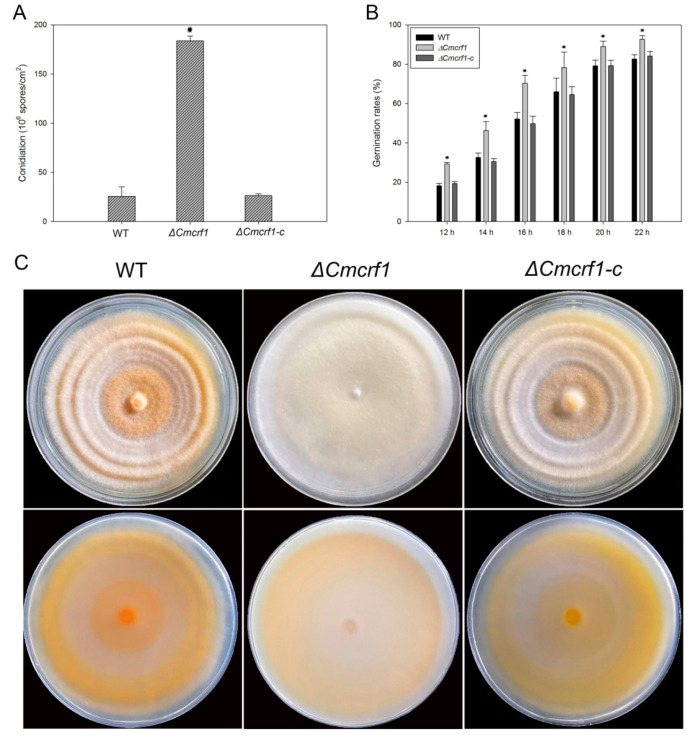
Growth characteristics of the WT strain and the mutants. (**A**) Conidiation of the WT strain and the mutants. (**B**) Conidial germination rates of the WT strain and the mutants. (**C**) Colony characteristics of the WT strain and the mutants. All of the strains were cultivated in the dark at 25 °C for 20 days and then exposed to light for 12 h. The error bars represent the standard deviations of three replicates. The data were statistically analyzed using Student’s *t*-test. Asterisks indicate significant differences between the WT strain and the *ΔCmcrf1* mutant (*p* < 0.05).

The growth characteristics of the WT strain, the *ΔCmcrf1* mutant, and the complemented mutant *ΔCmcrf1c* were performed on PDA at 25 °C for 3 weeks. There were no significant differences between the WT strain and the *ΔCmcrf1* mutant in terms of radial growth (Figure S3A). However, conidial production was seven-fold higher in the *ΔCmcrf1* mutant than in the WT strain (Figure 4A). The conidiation was restored to that of the WT strain when *Cmcrf1* was reintroduced to the null mutant. Furthermore, the conidial germination rates were analyzed to investigate whether *Cmcrf1* is required for conidial germination. The conidial suspensions of all of the *C. militaris* strains were inoculated on slides, which were then incubated in a moisture chamber at 25 °C. The conidia began to germinate at 12 h after inoculation. From 12 hpi to 22 hpi, the germination rate of the *ΔCmcrf1* mutant was significantly higher than that of the WT strain (Figure 4B).

To determine whether *Cmcrf1* affects the morphology of *C. militaris*, all of the strains were cultivated at 25 °C for 20 days in the dark. When the cultivated plates were transferred from dark to light conditions, the colonies of the *ΔCmcrf1* mutant remained white to light yellow, while those of the WT strain and the complemented mutant changed to yellow-orange after 12 h of light exposure (Figure 4C).

Furthermore, the carbon source utilization of all of the strains was examined on MM containing various carbon sources (Figure S3B). The results revealed that no significant differences were found between the carbon source utilization of the *ΔCmcrf1* mutant and that of the WT strain.

### 3.4. Effect of *Cmcrf1* on Cell-Wall Integrity

**Figure 5:**
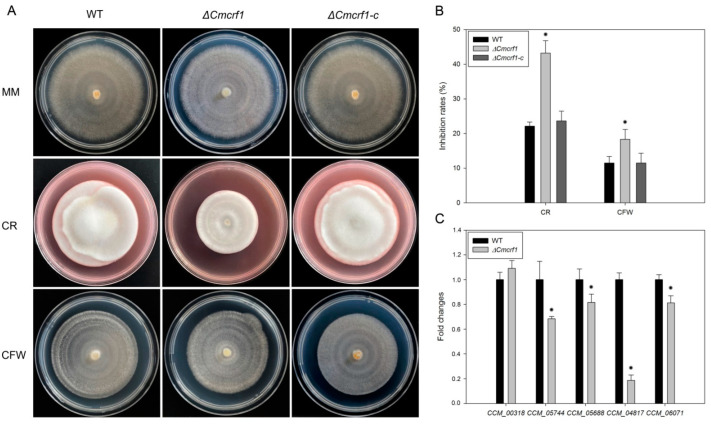
Sensitivity of the WT strain and the mutants to the cell-wall-perturbing chemicals. (**A**) Growth of all of the strains on minimal medium (MM) plates supplemented with 200 mg/L CR and 200 mg/L CFW. (**B**) The growth inhibition rate of mycelia in MM supplemented with CR and CFW. (**C**) Transcription abundance of genes encoding chitin synthases in the WT strain and the *ΔCmcrf1* mutant. The error bars represent the standard deviations of three replicates. The data were statistically analyzed using Student’s *t*-test. Asterisks indicate significant differences between the WT strain and the *ΔCmcrf1* mutant (*p* < 0.05).

The fungal cell wall is vital to the growth, survival, pathogenesis, and morphogenesis of a fungus, as it provides a protective barrier against a wide range of environmental stressors [32]. To examine effect of *Cmcrf1* on cell wall integrity, the sensitivity of the WT strain, the *ΔCmcrf1* mutant, and the complemented *ΔCmcrf1c* mutant to CFW and CR was compared. While all of the strains exhibited reduced growth in the presence of CFW or CR (Figure 5A,B), the growth inhibition rate of the *ΔCmcrf1* mutant was higher than that of the WT strain, indicating that *Cmcrf1* deletion increases hypersensitivity to substances that interfere with fungal cell-wall synthesis.

Chitin is one of the major components of fungal cell wall, which is synthesized by chitin synthases in ascomycetes. The transcription levels of five genes encoding chitin synthases (*CCM_00318*, *CCM_05744*, *CCM_05688*, *CCM_04817*, and *CCM_06071*) were examined in the WT strain and the *ΔCmcrf1* mutant. As seen in Figure 5C, transcript abundance of four chitin synthase genes were significantly decreased, whereas the transcript abundance of one chitin synthase gene *CCM_00318* was not altered in the *ΔCmcrf1* mutant. The results suggested that *Cmcrf1* is involved in cell wall synthesis, which further influences cell wall integrity via affecting the expression of genes that encode chitin synthases.

### 3.5. The *ΔCmcrf1* Mutant Showed Defective Development of Fruiting Bodies

**Figure 6:**
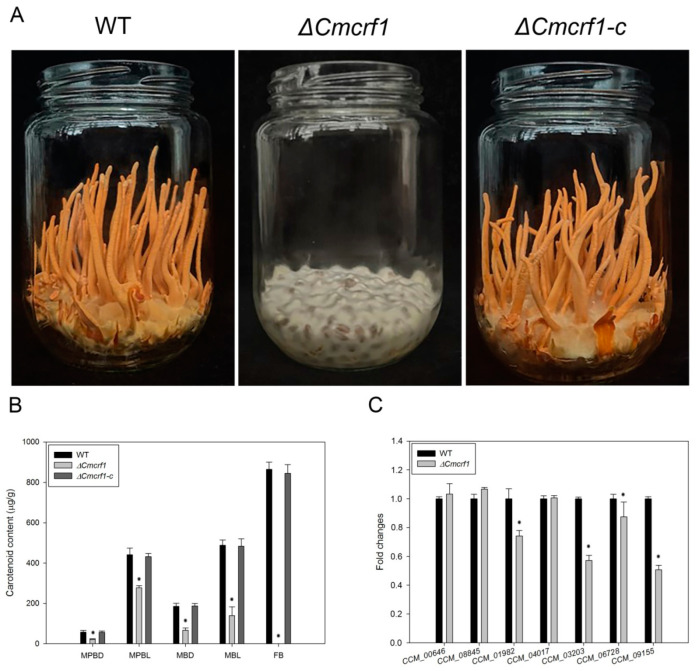
Fruiting body production and carotenoid concentration of the WT and mutant strains. (**A**) Fruiting body production of strain. All of the strains were cultivated on wheat-seed medium for 45 days. (**B**) Carotenoid concentration of the WT strain and the *ΔCmcrf1* mutant. MPBD, mycelia grown in PDB in the dark; MPBL, mycelia grown in PDB in the dark and then exposed to light for 12 h; MBD, mycelia grown in bottles in the dark; MBL, mycelia grown in bottles in the dark and then exposed to light for 12 h; FB, fruiting body. (**C**) Relative expression levels of seven genes that participated in carotenoids biosynthesis pathway. The error bars indicate the standard deviations of three replicates. The data were analyzed using Student’s *t*-test. Asterisks indicate significant differences between the WT strain and the *ΔCmcrf1* mutant (*p* < 0.05).

The fruiting body development of the WT strain, the *ΔCmcrf1* mutant, and the *ΔCmcrf1c* mutant on wheat-grain medium was compared. The WT strain produced normal, orange-colored fruiting bodies. However, the mycelia of the *ΔCmcrf1* mutant were white to light yellow and unable to produce even primordia for fruiting body formation (Figure 6A). The capacity to produce fruiting bodies was recovered in *ΔCmcrf1c*. These results indicate that *Cmcrf1* plays an essential role in fruiting body development in *C. militaris*.

### 3.6. *Cmcrf1* Affects the Carotenoid Content of C. militaris

For the determination of carotenoid content, the WT strain, the *ΔCmcrf1* mutant, and the *ΔCmcrf1c* mutant were cultivated on PDA plates, in potato dextrose broth in flasks, and on wheat-seed medium under different light-exposure conditions. As seen in Figure 6B, the carotenoid content in the hyphae of the *ΔCmcrf1* mutant was up to 3.51-fold lower than that in the hyphae of the WT strain after 12 h of light exposure. Moreover, the carotenoid content in the primordia and fruiting bodies of the *ΔCmcrf1* mutant could not be determined, as it did not form either of these structures. These results are consistent with the observed colony colors, indicating that *Cmcrf1* influences pigment synthesis and is thus a regulatory genetic factor in *C. militaris*.

Recently, a putative carotenoid biosynthetic pathway was proposed, and seven genes were reported to participating in this putative pathway [18]. We then examined the expression levels of these seven genes (*CCM_00646*, *CCM_08845*, *CCM_01982*, *CCM_04017*, *CCM_03203*, *CCM_06728*, and *CCM_09155*) by qRT-PCR. The expression levels of four genes (*CCM_01982*, *CCM_03203*, *CCM_06728*, and *CCM_09155*) were significantly down-regulated, while the expression levels of the other three genes (*CCM_00646*, *CCM_08845*, and *CCM_04017*) were not altered in the *ΔCmcrf1* mutant (Figure 6C). The results revealed that *Cmcrf1* has a role in carotenoids biosynthesis through influencing the expression of genes involved in this putative carotenoid biosynthetic pathway.

## 4. Discussion

The Zn_2_Cys_6_ zinc cluster proteins are a family of transcription factors found exclusively in fungi [33]. They function as transcriptional regulators and are involved in a wide variety of biological processes, including asexual and sexual development, secondary metabolite biosynthesis, carbon and nitrogen metabolism, stress responses, pleiotropic drug resistance, and fungal pathogenesis [34,35,36,37,38]. However, the functions of Zn_2_Cys_6_ TFs in different fungi are diverse. In the rice blast fungus *Magnaporthe oryzae*, the functions of 104 genes encoding Zn_2_Cys_6_ transcription factors were systematically analyzed. Deletion of these genes resulted in phenotypic changes in fungal development and pathogenicity, such as changes in conidiation, mycelial growth, pigmentation, appressorium formation, response to abiotic stresses, and colony morphology [39]. In the model filamentous fungus *N. crassa*, 135 genes encoding Zn_2_Cys_6_ transcription factors were found to be involved in basal hyphal growth, asexual sporulation, and sexual development [40]. When comparing the phenotypes of the Zn_2_Cys_6_ TFs homologs of *M. oryzae* and *N. crassa*, the results seems to be consistent with those previously reported that evolve differently in how to regulate fungal growth and asexual development rather than maintaining the same function in different fungi [41]. In this study, a color-changing-defective mutant T111 was isolated using T-DNA insertional mutagenesis, and *Cmcrf1*, which putatively encodes a Zn_2_Cys_6_ transcription factor, was identified in *C. militaris*. Our results showed that compared with the *C. militaris* WT strain, the *ΔCmcrf1* mutant exhibited excessive conidiation, a decreased conidial germination rate, and a higher sensitivity to cell-wall-perturbing agents. Furthermore, compared with the WT strain, the two most prominent important phenotypic characteristics of the *ΔCmcrf1* mutant were its drastically reduced carotenoid content and halted fruiting body formation. These results indicate that *Cmcrf1* is involved in fungal development, stress responses, secondary metabolite biosynthesis, and fruiting body development in *C. militaris*.

Carotenoids are massively accumulated in the mycelia and fruiting bodies of *C. militaris* upon light irradiation during the fungal cultivation process. However, little is known about the genetic regulatory mechanism of carotenoid biosynthesis in *C. militaris* [9,10,11]. In this study, the most remarkable phenotypic differences between the WT strain and the *ΔCmcrf1* mutant was that the *ΔCmcrf1* mutant contained significantly lower concentrations of carotenoids than the WT strain. Recently, a putative carotenoid biosynthetic pathway was depicted by integrating with DEGs and genome-scale metabolic network [18]. Seven genes were reported to participate in this putative pathway. In the *ΔCmcrf1* mutant, the transcript abundance of four genes were significantly down-regulated, while the other three genes were similar with the WT strain, indicating that reduction in carotenoids contents are probably led by the comprehensive effect of the decreased expression level of genes involved in the putative carotenoid biosynthetic pathway.

*Cmcrf1* is also involved in fungal development and fruiting body development. To date, seven photoreceptors have been identified in *C. militaris,* and three photoreceptors—*Cmwc-1*, *Cmvvd*, and *Cmcry-DASH*—were reported to regulate fungal development, such as conidiation and fruiting body formation, in *C. militaris* [15,16,17]. In addition, all three genes affect carotenoid production. Genes participating in other metabolic pathways, such as *Cmhyd1* encoding a class II hydrophobin and *Cmfhp* encoding an NO dioxygenase, have also been reported to regulate carotenoid accumulation and fungal development simultaneously [12,13]. The regulation of growth and development in response to environmental stimuli is a basic biological process in all organisms. Many filamentous fungi synthesize carotenoid pigments in response to several internal and external cues, including fungal development and light exposure [42]. These findings suggest that genes involved in carotenoid synthesis seem to also participate in the development of *C. militaris*. Nevertheless, whether carotenoid production plays an important biological role in fungal development in *C. militaris* and that of filamentous fungi in general remains to be determined.

Chitin is an essential component of fungal cell wall, and its synthesis depends on the activity of chitin synthases [43]. In this study, the *ΔCmcrf1* mutant showed defects in cell wall integrity and a higher sensitivity to cell-wall-perturbing agents CR and CFW than the WT strain. Previous studies reported that chitin synthases coding genes play crucial roles in the growth, conidiation, and cell wall integrity through regulating chitin contents in phytopathogenic fungi [44,45,46,47]. In the present study, the expression levels of four chitin synthases were significantly down-regulated in the *ΔCmcrf1* mutant compared to the WT strain, implying that *Cmcrf1* probably directly or indirectly regulates chitin synthesis, which further influences the response to different cell wall stressors.

## 5. Conclusions

Our study found that *Cmcrf1* is involved in carotenoid biosynthesis and fruiting body development in *C. militaris*, as it negatively regulates conidiation and cell wall integrity. These findings broaden our understanding of the functions of Zn_2_Cys_6_-type transcription factors in this edible and medicinal fungus. Future studies are warranted to further elucidate how carotenoid biosynthesis is regulated in *C. militaris* and to determine the relationship between fungal development and the role of *Cmcrf1* in carotenoid biosynthesis.

## Data Availability

Not applicable.

## Supplementary Materials

The following supporting information can be downloaded at: https://www.mdpi.com/article/10.3390/biology11101535/s1, Figure S1: Construction of the knockout vector pCAMBIA1300-yprKO (A) and the complementary vector pCAMBIA3301-yprC (B); Figure S2: Amino acid sequence alignment and phylogenetic analysis of *Cmcrf1* with the homologues from other fungal species; Figure S3: Growth of *C. militaris* strains on different carbon sources; Table S1: Primers used in this study.
