# Supplementary material for: Cmcrf1, a Putative Zn2Cys6 Fungal Transcription Factor, Is Involved in Conidiation, Carotenoid Production, and Fruiting Body Development in Cordyceps militaris
Source: Biology (Basel). 2022 Oct 19;11(10):1535. doi: 10.3390/biology11101535 (PMC9598893; doi:10.3390/biology11101535)
Supplement: Supplementary file 1 [file biology-11-01535-s001.zip › biology-1923453-supplementary.pdf]

Article

Cmcrf1, a Putative Zn2Cys6 Fungal Transcription Factor, Is Involved in Conidiation, Carotenoid Production and Fruiting Body Development in *Cordyceps militaris*

Ronglin He [1,2,*,†], Lin Zhang [1,2,3,†], Jinling Lan [3], Shengjie Mei [1,2,3] and Yu Li [2,3,*]

[1] Tianjin Institute of Industrial Biotechnology, Chinese Academy of Sciences, Tianjin 300308, China
[2] National Center of Technology Innovation for Synthetic Biology, Tianjin 300308, China
[3] College of Plant Protection, Jilin Agricultural University, Changchun 130118, China
* Correspondence: he_rl@tib.cas.cn (R.H.); liyu@tib.cas.cn (Y.L.); Tel./Fax: +86-22-24828751 (R.H. & Y.L.)
† These authors contributed equally to this work.

Simple summary: *Cordyceps militaris* produce a wide variety of bioactive components, such as cordycepic acid, cordycepin, polysaccharides, pentostatin, ergosterol, and carotenoids. In particular, Natural caotenoids from *C. militaris* are attracting increasing attention in human healthy and food coloring. Investigating the genetic regulatory mechanism of carotenoid biosynthesis will help to increase the carotenoid content of *C. militaris* through genetically engineering. This study focuses on the role of a putative Zn_2Cys_6 fungal transcription factor *Cmcrf1* on carotenoid biosynthesis and fruiting body formation. Deletion of *Cmcrf1* exhibited drastically reduced carotenoid biosynthesis and failed to generate fruiting bodies. In addition, the *ΔCmcrf1* mutant exhibited significantly increased conidiation and increased hypersensitivity to cell wall-perturbing agents. This study is helpful to deepen our knowledge of the regulatory mechanism of carotenoid biosynthesis in *C. militaris*.

Abstract: *Cordyceps militaris* is a high-value medicinal and edible fungus that produces many bioactive compounds, including carotenoid and thus improving the carotenoid productivity of *C. militaris* will increase its commercial value. However, little is known about the genetic regulatory mechanism of carotenoid biosynthesis in *C. militaris*. To further understanding the regulatory mechanism of carotenoid biosynthesis, we performed a large-scale screen of T-DNA insertional mutant library and identified a defective mutant, denoted T111, whose colonies did not change color from white to yellow upon exposure to light. Mutation analysis confirmed that a single T-DNA insertion occurred in the gene encoding a 695-amino acid putative fungal-specific transcription factor with a predicted Zn_2Cys_6 binuclear cluster DNA-binding domain found uniquely in fungi. Targeted deletion of this gene, denoted *C. militaris* carotenogenesis regulatory factor 1 (*Cmcrf1*), generated the *ΔCmcrf1* mutant that exhibited drastically reduced carotenoid biosynthesis and failed to generate fruiting bodies. In addition, the *ΔCmcrf1* mutant showed significantly increased conidiation and increased hypersensitivity to cell wall-perturbing agents compared with the wild-type strain. However, the *Cmcrf1* gene did not have an impact on the mycelia growth of *C. militaris*. These results show that *Cmcrf1* is involved in carotenoid biosynthesis and is required for conidiation and fruiting body formation in *C. militaris*.

Keywords: T-DNA insertional mutant; fungal development; carotenoid biosynthesis; edible and medicinal fungi

Supplementary Materials: The following supporting information can be downloaded at: www.mdpi.com/xxx/s1, Figure S1: Construction of the knockout vector pCAMBIA1300-yprKO and the complementary vector pCAMBIA3301-yprC; Figure S2: Amino acid sequence alignment and phylogenetic analysis of CmCRF1 with the homologues from other fungal species; Figure S3: Growth of *C. militaris* strains on different carbon sources; Table S1: Primers used in this study.

Table S1. Primers used in this study.

Primers	Sequences (5'-3') *
CRF15F	GGCAGCCCCTTACCCGACAACCT
CRF15R	TAAACCAAAATAGCATTGATGTGTTGACCTCCACCGCGGATACACCAC AAAACAAAA
CRF13F	AGCACTCGTCCGAGGGCAAAGGAATAGAGTAGATACGGCAACTTGAA GGGAAATACG
CRF13R	AGAGCGACAAACCTGCCCCAAGAG
hphF	GGAGGTCAACACATCAATG
hphR	CTACTCTATTCCTTTGCCCTCG
CRF1checkF	CCAGCGGCCCTACGACACAAC
CRF1checkR	CCGGCGGTACAATAAGATGACAGA
CRF1HBF	TAgaattcGGCAGCCCCTTACCCGACAACCT
CRF1HBR	TTggatccCGGCGCGGAGAAGATCAACATAAA
CRF1qPCRF	GCAGCCTGGTCACTCTTATT
CRF1qPCRR	GCTGAGGTATTGGCAGTAGTAG
cCRF1F	ATGGCTACGCGAGTGCCTCTCGGA
cCRF1R	TTATGCTGTTGCCATGGCCAAATC
TEF1qPCRF	GTCAAGGAAATCCGTCGTGGTAA
TEF1qPCRR	GCAGGCGATGTGAGCAGTGTG
LAD1	ACGATGGACTCCAGAGCGGCCGCVNVNNNGGAA
LAD2	ACGATGGACTCCAGAGCGGCCGCBNBNNNGGTT
LAD3	ACGATGGACTCCAGAGCGGCCGCVVNVNNNCCAA
LAD4	ACGATGGACTCCAGAGCGGCCGCBDNBNNNCGGT
AC1	ACGATGGACTCCAGAG
RB-0	CGTGACTGGGAAAACCCTGGCGTT
RB-1	ACGATGGACTCCAGAGCGACCCAACTTAATCGCCTTGCAGCACATC
RB-2	GAAGAGGCCCGCACCGATCGCCCTT

* Low letters indicated the restriction enzyme sites.

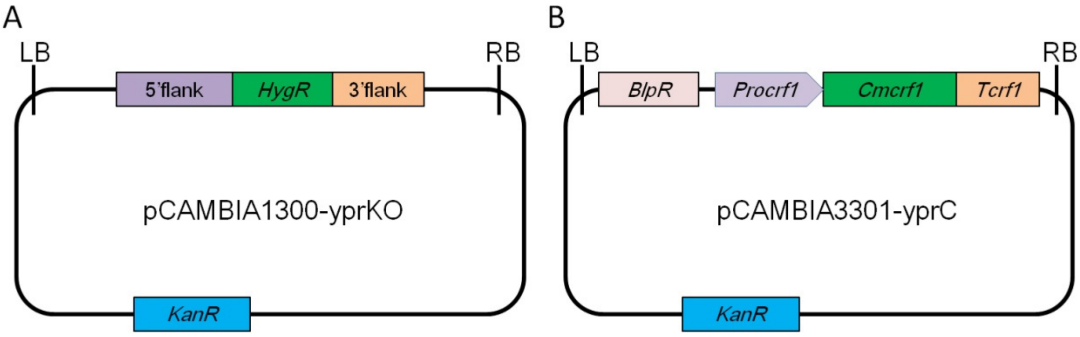

Figure S1. Construction of the knockout vector pCAMBIA1300-yprKO (A) and the complementary vector pCAMBIA3301-yprC (B). *HygR*, hygromycin B phosphotransferase gene; *BlpR*, phosphinothricin acetyltransferase gene; *KanR*, kanamycin gene. *Procrf1*, promoter of the *Cmcrf1* gene; *Tcrf1*, terminator of the *Cmcrf1* gene.

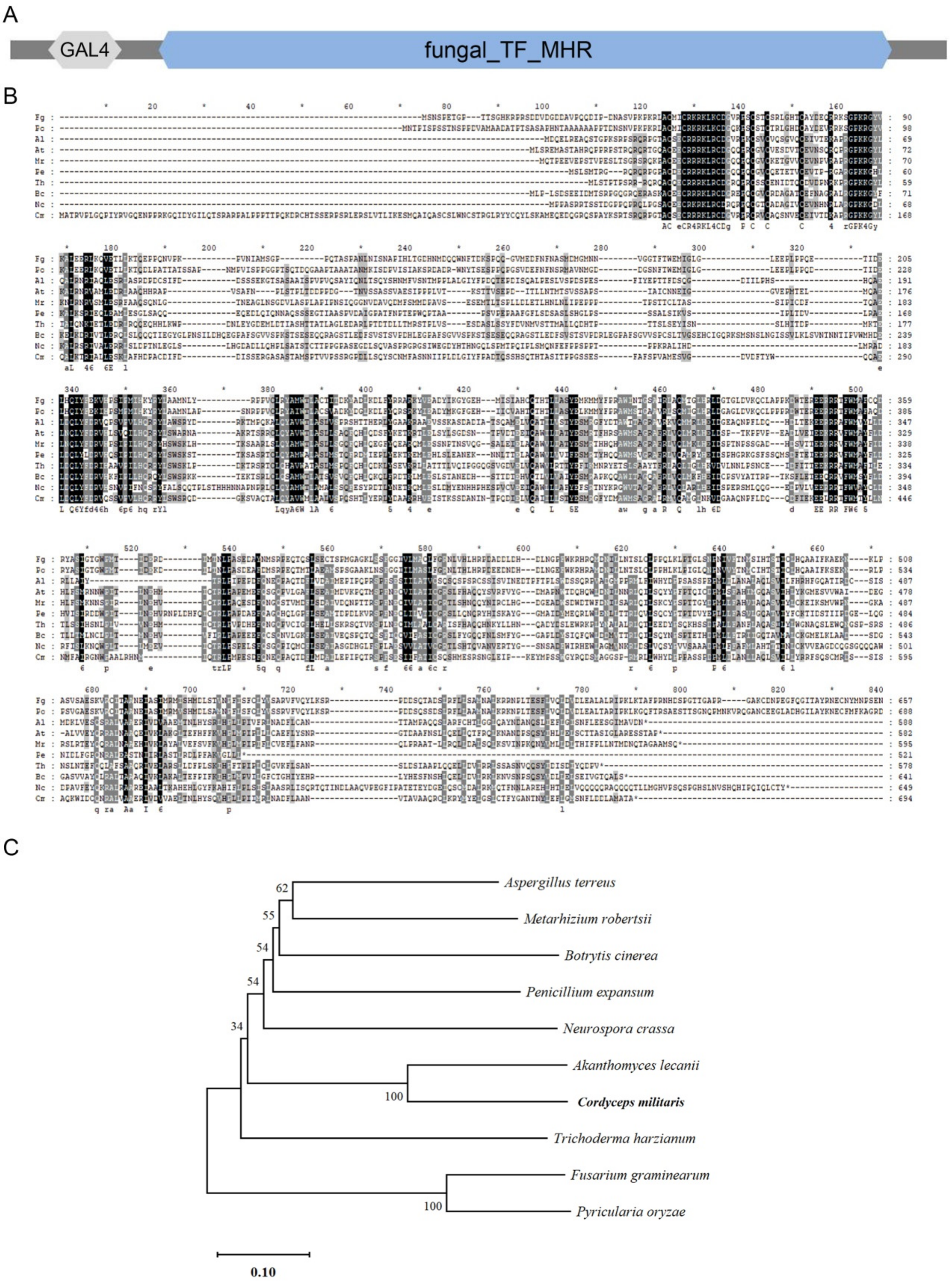

Figure S2. Amino acid sequence alignment and phylogenetic analysis of CmCRF1 with the homologues from other fungal species. (A) Prediction of the functional domains of CmCRF1 using the Conserved Domain Database (http://www.ncbi.nlm.nih.gov/Structure/cdd/cdd.shtml). GAL4, GAL4-like Zn2Cys6 binuclear cluster DNA-binding domain; fungal TF-MHR, fungal transcription factor regulatory middle homology region. (B) Alignment of the predicted amino acid sequence of CmCRF1 with its homologs from other fungi. Amino acid sequences were aligned using the CLUSTALW program (http://www.ebi.ac.uk/clustalw). Identical amino acids are colored black, and similar amino acids are colored gray. Fg, *Fusarium graminearum*; Po, *Pyricularia oryzae*; Al, *Akanthomyces lecanii*; At, *Aspergillus terreus*; Mr, *Metarhizium robertsii*; Pe, *Penicillium expansum*; Th, *Trichoderma harzianum*; Bc, *Botrytis cinerea*; Nc, *Neurospora crassa*; Cm, *Cordyceps militaris*. (C) Phylogenetic analysis of CmCRF1. The tree was reconstructed using a neighbor-joining method after alignment with the CLUSTALW algorithm using MEGA4 program. Numbers at the nodes in the

rooted tree represent bootstrapping values for 1,000 replications. Accession numbers are as follows: *F. graminearum* (PCD22760.1), *P. oryzae* (XP_003710596.1), *A. lecanii* (OAQ97806.1), *A. terreus* (KAG2418079.1), *M. robertsii* (EXU98962.1), *P. expansum* (KGO48705.1), *T. harzianum* (XP_024774947.1), *B. cinerea* (XP_024547559.1), *N. crassa* (XM_954030.2), and *C. militaris* (XP_006673201.1).

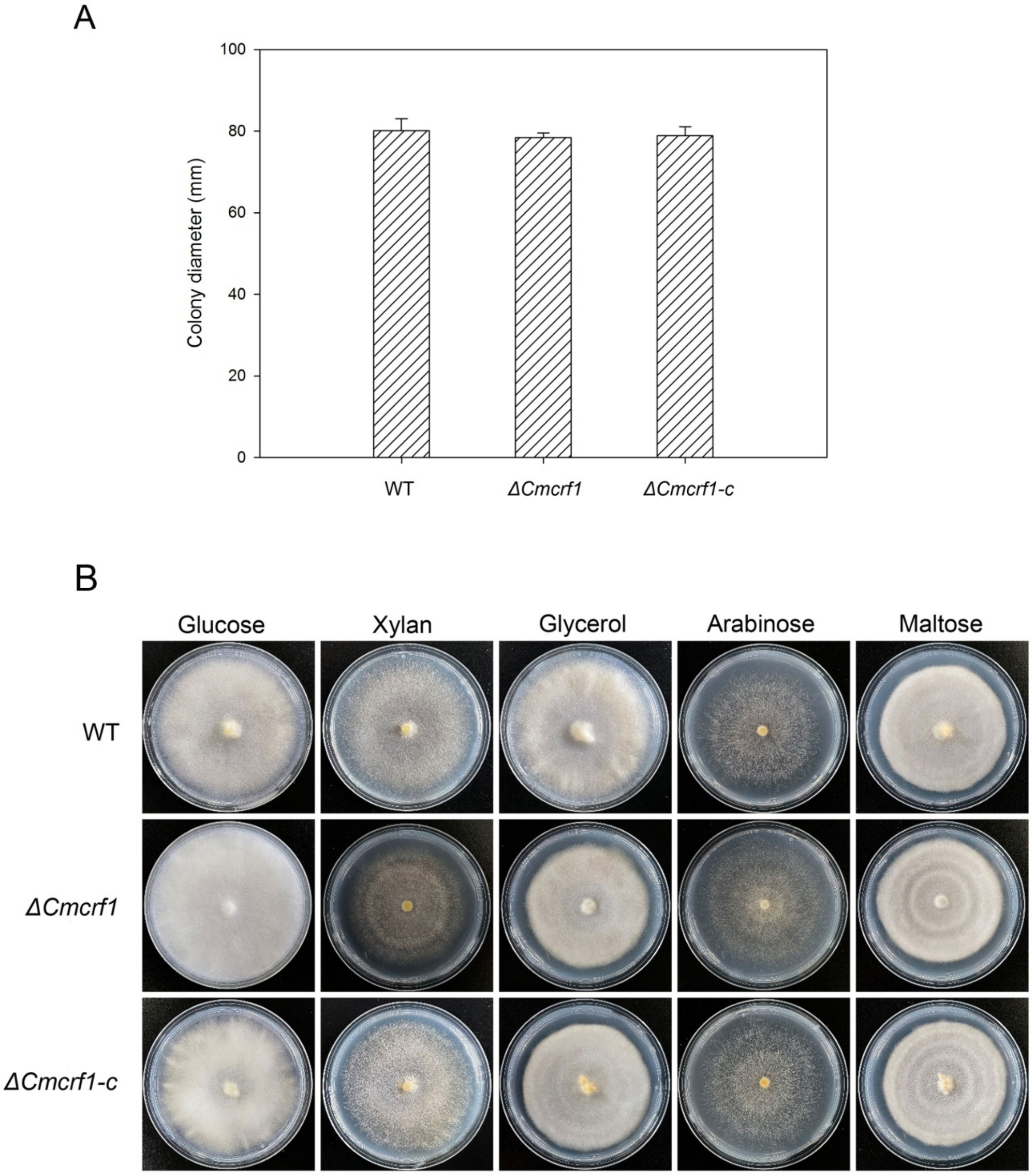

Figure S3. Growth of *C. militaris* strains on different carbon sources. (A) Colony diameters of the WT strain, the *ΔCmcrf1* mutant, and the complemented mutant *ΔCmcrf1-c* on PDA plates. The colony diameters of all of the tested strains were measured after 20 days of cultivation at 25°C. (B) Morphology of all strains grown on MM plates containing different carbon sources. All strains were grown on MM supplemented with 1% glucose, xylan, glycerol, arabinose, or maltose at 25°C for 20 days.
